# Supplementary material for: Physicochemical and Sensorial Evaluation of Meat Analogues Produced from Dry-Fractionated Pea and Oat Proteins
Source: Foods. 2020 Nov 27;9(12):1754. doi: 10.3390/foods9121754 (PMC7760771; doi:10.3390/foods9121754)
Supplement: Supplementary file 1 [file foods-09-01754-s001.pdf]

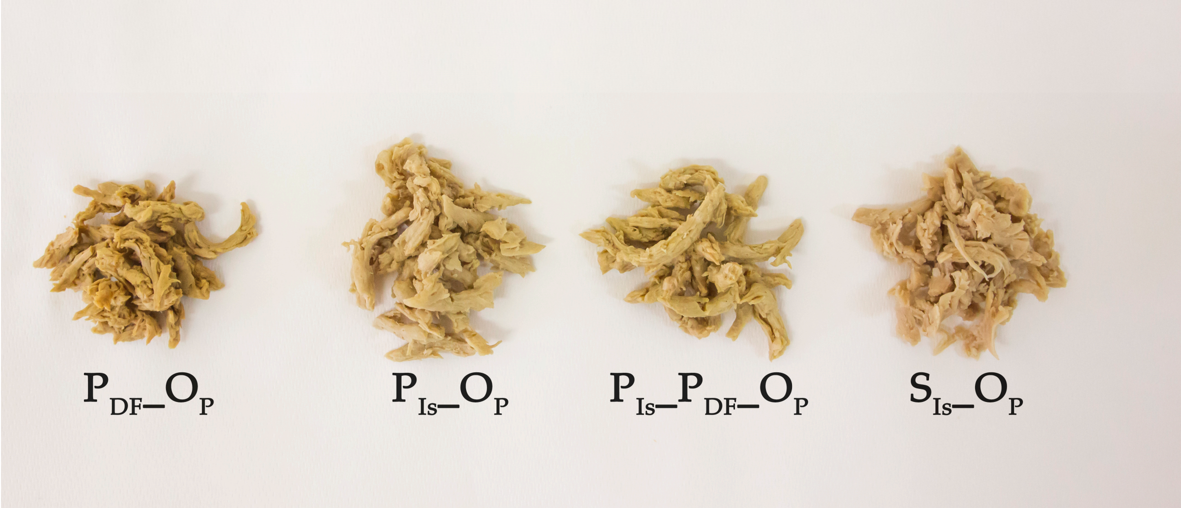

Figure 1. Rehydrated meat analogues produced by low-moisture extrusion cooking. PDF_OP: dry-fractionated pea protein and oat protein; PIs_OP: pea protein isolates and oat protein; PDF_PIs_OP: dry-fractionated pea protein, pea protein isolate, and oat protein; SIs_OP: soy isolates and oat protein.
